# Screening, brief intervention, and referral to treatment (SBIRT) in an emergency department: three-month outcomes of a randomized controlled clinical trial among Mexican-origin young adults

**DOI:** 10.1186/1940-0640-8-S1-A17

**Published:** 2013-09-04

**Authors:** Cheryl J Cherpitel, Robert Woolard, Yu Ye, Jason Bond, Ed Bernstein, Judith Bernstein, Susana Villalobos, Rebeca Ramos

**Affiliations:** 1 Public Health Institute, Alcohol Research Group, Emeryville, CA, USA; 2 Texas Tech University, Health Science Center, El Paso, TX, USA; 3 Boston University School of Medicine, Boston, MA, USA; 4 Alliance of Border Collaboratives, El Paso, TX, USA

## 

A randomized controlled trial of screening, brief intervention, and referral to treatment (SBIRT) for drinking and related problems among at-risk and dependent drinkers was conducted in an emergency department (ED) at the US-Mexico border among Mexican-origin young adults. Data collection over a period of 17 months resulted in 698 patients recruited into the study and randomized to one of three conditions: screened only (n=78), assessed (n=310), and intervention (n=310). Patients in the assessment (77%) and intervention (72%) conditions were blindly reassessed at three months via one telephone interview. No difference was found in baseline demographic or drinking characteristics between the assessment intervention groups. At follow-up, the intervention group showed significantly greater decreases in five of six outcome variables: number of drinking days per week, average number of drinks and per drinking day, maximum number of drinks, RAPS4 score as an indicator of alcohol dependence and number of negative consequences related to drinking. Using analysis of covariance to control for baseline measures, the intervention group was lower on all drinking and problem measures than the assessment group, and significantly so on four of the measures. When the interaction of intervention by injury status (injury vs. non-injury), drinking within six hours prior to the event, causal attribution of the event to drinking and risk taking disposition were examined, only causal attribution had a significant effect for two of the problem measures – the intervention effect for the RAPS4 and negative consequences was greater for those who believed their injury or illness was related to their drinking. Findings here suggest that brief intervention was effective in this population compared to assessment at three month follow-up, and may be most effective for those linking the reason for their ED visit to their drinking.

